# Maternal probiotic and prebiotic supplementation on glucose metabolism in pregnant women and their offspring: effects and related mechanisms

**DOI:** 10.3389/fmicb.2026.1782361

**Published:** 2026-03-10

**Authors:** Hanmo Lin, Chuhan Shao, Jie Yu, Haiyan Chen, Yaolin Ren, Jing Ren, Yuan Zeng, Yifan Wu, Qian Zhang, Xinhua Xiao

**Affiliations:** Key Laboratory of Endocrinology, Ministry of Health, Department of Endocrinology, Peking Union Medical College Hospital, Peking Union Medical College, Chinese Academy of Medical Sciences, Beijing, China

**Keywords:** diabetes, epigenetics, glucose metabolism, gut microbiota, offspring, prebiotics, probiotics

## Abstract

**Introduction:**

The global diabetes epidemic has brought gestational diabetes mellitus (GDM) and its long-term impacts on maternal-child health into sharp focus. Emerging evidence indicates that early-life metabolic programing, mediated significantly by gut microbiota, profoundly influences offspring glucose homeostasis. Notably, microbial-targeted nutritional interventions, including probiotic and prebiotic supplementation, have considerable potential as innovative therapeutic approaches. These strategies may effectively prevent intergenerational transmission of metabolic diseases by improving glucose metabolism in both mother and offspring.

**Methods:**

This narrative review synthesizes evidence from clinical trials and animal studies investigating the effects of maternal probiotic and prebiotic supplementation on glucose metabolism. We searched and analyzed literature focusing on glycemic outcomes in pregnant women with or without GDM and their offspring, as well as studies exploring underlying mechanisms including gut microbiota modulation, metabolite production, inflammatory pathways, and epigenetic regulation.

**Results:**

Clinical and animal studies have shown that probiotics and prebiotics can significantly alleviate metabolic parameters such as elevated fasting glucose and insulin resistance in patients with GDM, but their preventive effect on the incidence of GDM is unclear. In addition, maternal supplementation with probiotics or prebiotics may positively affect glucose metabolism in offspring through multiple interconnected mechanisms, which include the modulation of intestinal microbial ecology, the increased generation of microbial- derived metabolites such as short-chain fatty acids (SCFAs), the mitigation of inflammatory responses, and epigenetic regulation (e.g., DNA methylation, lncRNA and miRNA modification).

**Discussion:**

Despite some heterogeneity in the results of existing studies, there is overall support for the therapeutic potential of probiotic and prebiotic interventions in optimizing metabolic outcomes for both maternal and pediatric populations. Future studies need to further define the optimal type, dose and timing of intervention for probiotics and prebiotics and explore precise intervention strategies on the basis of individual gut microbiota characteristics. In conclusion, probiotic and prebiotic supplementation during pregnancy and lactation may become an adjunctive tool to improve glucose metabolism in mothers and infants, resulting in innovative approaches for the primary prevention of metabolic diseases.

## 1 Introduction

The prevalence of type 2 diabetes has risen significantly globally in recent years, constituting a major public health challenge worldwide. According to the latest data from the International Diabetes Federation, in 2024, 589 million individuals (aged 20–79) currently live with diabetes, with projections suggesting that this figure may increase to 853 million by 2050. China’s diabetic population exceeds 118 million individuals, accounting for 22% of the total number of patients globally, making China one of the countries with the highest burden of diabetes in the world. Diabetes not only significantly elevates the risk of multiple systematic complications such as cardiovascular disorders, visual impairment, renal failure, infections and amputations (especially of the lower limbs), but also consumes 12% of the global health expenditure in 2024, approximately $1.015 trillion, underscoring the urgency of its prevention and early intervention.

Gestational diabetes mellitus (GDM) poses significant risks to both maternal and fetal health, with potential long-term implications. This condition increases the likelihood of adverse pregnancy outcomes, such as hypertensive disorders (e.g., preeclampsia), fetal macrosomia (excessive birth weight), and delivery complications, including obstructed labor. It is estimated that 1 in 5 live births (23 million) in 2024 were affected by hyperglycemia during pregnancy, and 1/6 live births were affected by gestational diabetes. These babies are at higher risk for a range of diabetes-related complications.

Emerging research has revealed the critical influence of early-life environmental factors on long-term metabolic outcomes. The Developmental Origins of Health and Disease (DOHaD) hypothesis posits that early-life environmental exposures can induce permanent effects on physiological structure and function through metabolic programing throughout the lifespan (Moore, 2017; Prentice, 2018; Reynolds et al., 2015). Maternal metabolic disturbances—including hyperglycemia, hyperlipidemia, and obesity—have been implicated as significant risk factors for offspring metabolic dysfunction later in life (Raghavan et al., 2017; Seet et al., 2015; Zambrano et al., 2016). Therefore, exploring effective interventions during early life is critical for disrupting the transgenerational cycle of metabolic disorders and modifying pathological developmental pathways.

Against this backdrop, using probiotics and prebiotics as microecological intervention strategies demonstrate potential value. Probiotics are live microorganisms that can benefit host health when given in sufficient quantities (Hill et al., 2014) and can act directly on the intestinal barrier, modulate immune responses, improve glucose tolerance, and restore the imbalanced state of the gut microbiota caused by various metabolic diseases. Prebiotics are non-digestible dietary components that can be selectively utilized by host microorganisms and provide health benefits (Gibson et al., 2017), mainly including oligofructose (FOS), inulin and galacto-oligosaccharides (GOS) (García-Montero et al., 2023). These bioactive constituents modulate the intestinal microbial ecology through the selective stimulation of commensal microorganisms, particularly taxa with demonstrated metabolic benefits, including *Bifidobacterium* and *Lactobacillus* species. Copious evidence suggests the benefits of probiotic and prebiotic supplementation in modulating glucose metabolism, with maternal supplementation during pregnancy shown to improve metabolic parameters in both mothers and offspring (Bernini et al., 2016; De Lorenzo et al., 2017; Hsieh et al., 2018). More significantly, the exploration of their mechanisms is increasingly in-depth, extending beyond mere microbial modulation to encompass multidimensional, multi-pathway systemic regulation. Studies have shown that probiotic or prebiotic supplementation plays a role mainly by regulating the composition of gut microbiota, promoting the production of short chain fatty acids (SCFAs) and other metabolites, reducing inflammatory response, and epigenetic regulation (such as DNA methylation, lncRNA and miRNA modification). By exploring the mechanism of probiotics or prebiotics, we can deepen our understanding of the regulation pathway of glucose metabolism, provide more accurate targets for the treatment of metabolic diseases in the future, and promote the exploration of individualized intervention strategies.

This review focuses on the effects of maternal probiotic and prebiotic supplementation on glucose metabolism in the mother and offspring and provides an overview of its possible mechanisms.

## 2 Gut microbiota: key regulators of glucose metabolism and intervention targets

### 2.1 Regulation mechanism of gut microbiota on glucose metabolism

Existing epidemiologic and physiologic-based findings suggest that the microbiota can mediate environmental effects on human health and disease. Most of the microbiota that inhabit the human body are located in the gut and include bacteria, fungi, archaea and viruses (Woo and Alenghat, 2022). The number of all gut microbial genomes in a healthy human individual is 10–100 times larger than the human genome (Tremaroli and Bäckhed, 2012). The gut microbiota has important functions in the human body such as barrier function, metabolic responses, nutritional effects and immune responses, especially with respect to metabolism. It can ferment dietary fiber and produce gas, short-chain fatty acids (SCFAs), organic acids, etc. SCFAs provide an additional source of energy for colonocytes and stimulate the secretion of gut hormones such as Glucagon like peptide-1 (GLP-1) to regulate glucose metabolism. Undigested proteins can be catabolized and metabolized by the gut microbiota to synthesize some essential amino acids, among which branched-chain amino acids (BCAAs) and tryptophan (Trp) metabolism can affect glucose homeostasis via peripheral serotonin (Li T. et al., 2024). In addition, the gut microbiota is involved in the establishment of the intestinal mucosal barrier, thereby prevent metabolic inflammation caused by endotoxin translocation.

Also, the gut microbiota can directly or indirectly alternate gut-brain axis signal, thus effecting the activation of nerve afferent and central nervous system (ENS), thereby exerting downstream effects on feeding behavior, energy homeostasis, glycemic control, and pancreatic β-cell function (Wachsmuth et al., 2022).

### 2.2 The gut microbiota in metabolic diseases

Emerging research increasingly implicates gut microbial dysbiosis as a significant contributor to the pathogenesis of metabolic disorders, particularly obesity and T2DM (Murugesan et al., 2025). Under the state of obesity and metabolic diseases, there is an imbalance in the gut microbiota (Zhang et al., 2013), which is manifested by an increased *Firmicutes*/*Bacteroidetes* ratio (Magne et al., 2020), the depletion in the composition of the butyrate-producing bacteria *Akkermansia*, *Faecalibacterium*, *Oscillibacter*, and *Alistipes* (Allin et al., 2018; Thingholm et al., 2019), the increase in a variety of opportunistic pathogens (Qin et al., 2012), as well as the decline in the α-diversity of gut microbiota (Yuan et al., 2023). This dysregulated configuration compromises intestinal barrier function and promotes metabolic endotoxemia, which is one of the major contributors to insulin resistance and diabetes pathogenesis. A human fecal microbiota transplantation study has confirmed that transferring feces from lean individuals to patients with metabolic syndrome can increase their gut microbial diversity and significantly improve insulin sensitivity (Vrieze et al., 2012). This directly proves the causal role of changes in gut microbiota in metabolic diseases. Therefore, in recent years, microbial-targeted therapeutic strategies have emerged as a promising research focus, with increasing evidence supporting their potential to ameliorate metabolic dysfunction through microbiota modulation.

### 2.3 Development of the gut microbiota early in life

The conventional viewpoint suggests that gastrointestinal microbial colonization initiates during parturition and is influenced by factors such as delivery route (vaginal vs. cesarean), diet, and medications. However, with several recent studies reporting the detection of microbial particles in umbilical cord blood, placenta, and amniotic fluid from healthy pregnancies by 16S rRNA sequencing, the traditional view of a sterile intrauterine environment has been shattered, and the offspring microbiota may be colonized by maternal bacteria prior to birth through transplacental transport (Senn et al., 2020). Unlike that of adults, the gut microbiota of infants and young children is unstable. Around approximately 3 years of age, children establish a stable microbiota similar to that of adults. Therefore, early changes in gut microbiota - due to maternal lifestyle, prenatal exposure, delivery methods, and feeding habits—can profoundly influence offspring development and metabolic health (De Filippo et al., 2010; Xue et al., 2022).

Extensive studies have demonstrated that patients with GDM exhibit significant gut microbiota dysbiosis, characterized by an elevated Firmicutes/Bacteroidetes (F/B) ratio, reduced abundance of beneficial bacteria, increased prevalence of opportunistic pathogens, and decreased α-diversity (Wang S. et al., 2024). This dysbiosis is closely associated with metabolic phenotypes such as insulin resistance, elevated fasting glucose, and increased inflammatory levels. Moreover, the maternal gut microbiota dysbiosis in GDM can be vertically transmitted, influencing the early-life establishment of the infant gut microbiota and contributing to an intergenerational transmission of metabolic risk (Collado et al., 2010; Galley et al., 2014). During the first 1–6 months postpartum, infants born to mothers with GDM display significantly lower abundance of *Akkermansia* and *Faecalibacterium*, higher abundance of *Romboutsia*, *Oscillibacter*, and *Lachnoclostridium* (Qin et al., 2022), and reduced α-diversity compared to healthy controls (Song Q. et al., 2023). These microbial differences are positively associated with infant BMI (Zhu et al., 2022) and insulin resistance index (Hasan et al., 2018).

### 2.4 Gut microbiota intervention strategies and mechanisms

Common gut microbiota interventions include probiotic and prebiotic supplementation. Probiotics are live microorganisms that benefit host health (Hill et al., 2014) and can act directly on the intestinal barrier to modulate the immune response, thereby improving glucose tolerance and potentially ameliorating obesity and diabetes by restoring gut dysbiosis; prebiotics are non-digestible dietary compounds that undergo selective fermentation by host microorganisms, mainly including oligofructose (FOS), inulin, and galactooligosaccharides (GOS) (García-Montero et al., 2023), thereby inducing quantitative and qualitative modifications in the intestinal microbial ecosystem that yield clinically relevant health improvements (Gibson et al., 2017). Probiotic and prebiotic interventions can lead to improvements in specific metabolic parameters, such as reductions in FBG (Fasting Blood Glucose) and HbA1c levels (Kim et al., 2018; Tabuchi et al., 2003; Tonucci et al., 2017). Additionally, synbiotics are mixture comprising live microorganisms and substrate(s) selectively utilized by host microorganisms, which confers a health benefit on the host (Swanson et al., 2020). These combined formulations may confer additive or even synergistic metabolic benefits.

Probiotics and prebiotics can improve glucose metabolism through several mechanisms. Firstly, they can increase the quantity of intestinal metabolites such as SCFAs (Falcinelli et al., 2016; Zartl et al., 2018), thereby increasing insulin sensitivity and inhibit hepatic gluconeogenesis. Secondly, they enhance intestinal barrier function and decrease systemic inflammation levels by reducing intestinal permeability (Anderson et al., 2010). Thirdly, they can stimulate the secretion of intestinal hormones such as glucagon-like peptide-1 (GLP-1), which promotes insulin release and suppresses appetite (Bjerg et al., 2014).

## 3 Effects of probiotic and prebiotic supplementation on glucose metabolism on self

### 3.1 Maternal prebiotic and probiotic interventions improve glucose metabolism in GDM

GDM is a prevalent form of pregnancy-related metabolic dysfunction, characterized by pregnancy-induced insulin resistance that exceeds the compensatory capacity of pancreatic β-cell function. The underlying pathophysiology involves a complex interplay of placental-derived diabetogenic hormones (including human placental lactogen, prolactin, and cortisol), chronic low-grade inflammation, and maternal genetic susceptibility (Alejandro et al., 2020). Metabolically, GDM manifests as fasting hyperglycemia, exaggerated postprandial glucose excursions, peripheral insulin resistance, and relative insulin deficiency (Sweeting et al., 2022). These disturbances not only increase maternal risk of pregnancy complications but also expose the developing fetus to an adverse intrauterine environment, leading to fetal hyperinsulinemia, excessive adiposity, and long-term metabolic programming (Raghavan et al., 2017; Seet et al., 2015; Zambrano et al., 2016).

Emerging evidence implicates gut microbial dysbiosis as a contributing factor in the pathogenesis of GDM. Therefore, targeted modulation of the gut microbiota through probiotic and prebiotic supplementation is a possible way to improve glucose metabolism in patients with GDM. Several randomized controlled trials (RCTs) (Ahmadi et al., 2016; Babadi et al., 2019; Dolatkhah et al., 2015; Jafarnejad et al., 2016; Karamali et al., 2016; Kijmanawat et al., 2019; Sahhaf Ebrahimi et al., 2019) and meta-analysis (Mu et al., 2023; Zheng et al., 2018) consistently showed that the probiotic intervention (such as *Bifidobacterium* and *Lactobacillus*) in pregnant women with GDM could significantly reduce FBG, FSI (Fasting Serum Insulin), HOMA-IR (Homeostatic Model Assessment of Insulin Resistance) and HbA1c levels (Table 1). There are no clinical studies on the impact of prebiotic supplementation on glucose metabolism in GDM patients. In animal studies, prebiotic (Miao et al., 2022; Zhang Q. et al., 2019) or probiotic (Castro-Rodríguez et al., 2020) supplementation has a clear effect on the improvement of metabolic parameters (such as OGTT AUC, FBG, FSI and HOMA-IR) of high-fat-diet pregnant mice (Table 2). Probiotic supplementation also independently reduced leptin levels in pregnant mice (Guo et al., 2018). In addition, Ahmadi et al. (2016) reported that an 8-week intervention with a synbiotic capsule containing *Lactobacillus acidophilus*, *Lactobacillus casei*, *Bifidobacterium bifidum*, and 800 mg inulin significantly reduced fasting serum insulin and HOMA-IR in women with GDM compared to placebo. Similarly, a meta-analysis by Mu et al. (2023) demonstrated that synbiotic supplementation significantly improved fasting plasma glucose, insulin resistance, and lipid profiles in GDM patients, with effect sizes comparable to or exceeding those observed with probiotics alone.

**TABLE 1:** Clinical studies of maternal prebiotic/probiotic supplementation and its primary maternal outcomes.

References	Study design	Subject population	Intervention	Primary maternal outcome
Mu et al. (2023)	Meta-analysis	GDM females	Probiotics/Synbiotics	Improve FPG, FSI, HOMA-IR and TC No significant effect on weight gain
Dolatkhah et al. (2015)	Randomized controlled trial (RCT)	GDM females	*Lactobacillus acidophilus* LA-5, *Bifidobacterium bifidum* BB-12, *Streptococcus thermophilus* STY-31 and *Lactobacillus bulgaricus* LBY-27	Reduce weight gain later in the study Improve FBS and HOMA-IR
Lindsay et al. (2015)	RCT	GDM females	*Lactobacillus salivarius* UCC118	No beneficial effect on glucose metabolism No significant effect on weight gain
Kijmanawat et al. (2019)	RCT	GDM females	*Bifidobacteria* and *Lactobacillus*	Improve FBG, FSI and HOMA-IR No significant difference in weight gain
Sahhaf Ebrahimi et al. (2019)	RCT	GDM females	*Acidophilus* and *Lactobacillus*	Improve FBG, PBG and HbA1c levels
Ahmadi et al. (2016)	RCT	GDM females	*Lactobacillus acidophilus*, *Lactobacillus casei*, and *Bifidobacterium bifidum* plus 800 mg inulin	Improve FSI and HOMA-IR
Babadi et al. (2019)	RCT	GDM females	*Lactobacillus acidophilus*, *Lactobacillus casei*, *Bifidobacterium bifidum*, *Lactobacillus fermentum*	Improve FBG, FSI and HOMA-IR, increase insulin sensitivity
Jafarnejad et al. (2016)	RCT	GDM females	VSL#3 (*Streptococcus thermophilus*, *Bifidobacterium breve*, *Bifidobacterium longum*, *Bifidobacterium infantis*, *Lactobacillus acidophilus*, *Lactobacillus plantarum*, *Lactobacillus paracasei*, and *Lactobacillus delbrueckii* subsp. *Bulgaricus*)	No significant difference in GWG Improve insulin levels and HOMA-IR without affecting FPG and HbA1c Decrease the level of IL-6, TNF-α and hs-CRP
Karamali et al. (2016)	RCT	GDM females	*Lactobacillus acidophilus*, *Typhimurium casei*, and *Bifidobacterium bifidum*	Decrease FBG and FSI, increase HOMA-IR and insulin sensitivity
Shahriari et al. (2021)	RCT	Pregnant women at high risk of GDM	*Lactobacillus acidophilus* LA1, *Bifidobacterium longum* sp54 cs, and *Bifidobacterium* sp9 cs	No reduction of GDM risk
Callaway et al. (2019)	RCT	Overweight and obese pregnant women	*Lactobacillus rhamnosus* and *Bifidobacterium animalis* subspecies lactis	No beneficial effects on glucose metabolism No significant effect on weight gain
Halkjær et al. (2020)	RCT	Obese pregnant women	Multi-strain probiotics Vivomixx^®^	No significant difference in GDM incidence and HbA1c concentration GWG was decreased, but the difference was not significant Increased α-diversity of gut microbiota with increased abundance of *Bifidobacterium*, *Lactobacillus*, and *S. salivarius*
Asgharian et al. (2020)	RCT	Overweight and obese pregnant women	*Lactobacillus acidophilus* La5 and *Bifidobacterium lactis* Bb12	Improve FPG and 2hOGTT No significant difference between GDM incidence and GWG
Pellonperä et al. (2019)	RCT	Overweight and obese pregnant women	*Lactobacillus ramosus* HN001 and *Bifidobacterium animalis* 420	No reduction in GDM incidence No significant improvement in glucose metabolism
Wan et al. (2023)	RCT	Healthy pregnant women	Galactooligosaccharide (GOS)	No significant differences in GDM incidence, GWG and glucose metabolism levels Specific increase in relative abundance of Paraprevotella and Dorea and decrease in relative abundance of *Lachnospiraceae* UCG_001
Wickens et al. (2017)	RCT	Pregnant women with a personal or partner history of atopic disease	*Lactobacillus rhamnosus* HN001	Reduce incidence of GDM and improve FBG No significant effect on weight gain
Jamilian et al. (2016)	RCT	Healthy pregnant women	*Lactobacillus acidophilus*, *Lactobacillus casei*, *Bifidobacterium bifidum*	Reduce FSI, improve HOMA-IR and QUICKI
Masulli et al. (2020)	Systematic review and Meta-analysis	Pregnant women	Probiotics	No reduction in GDM incidence, no significant reduction in FPG
Davidson et al. (2021)	Cochrane systematic reviews	Pregnant women	Probiotics	No prophylactic effect on GDM
Taylor et al. (2017)	Systematic review and Meta-analysis	GDM women	Probiotics	No significant effect on FBG, significant reduction in HOMA-IR No significant effect on weight gain
Zheng et al. (2018)	Meta-analysis	Pregnant women	Probiotics	For pregnant women with GDM, significant improvement in FBG and HOMA-IR. For healthy pregnant women, significant improvement in FSI and HOMA-IR
Chen et al. (2019)	RCT	Healthy pregnant women	*Bifidobacterium longum*, *Lactobacillus bulgaricus* and *Streptococcus thermophilus*	No change in the relative abundance of *Bifidobacterium*, *Lactobacillus* and *Streptococcus thermophilus* Significantly lower levels of Turicibacter and Phascolarctobacterium
Shadid et al. (2007)	RCT	Healthy pregnant women	Galactose (GOS) and long chain oligofructose (lcFOS)	Increased abundance of bifidobacteria
Jinno et al. (2017)	RCT	Healthy pregnant women	Fructooligosaccharides (FOS)	Increased abundance of bifidobacteria
Jones et al. (2024)	RCT	Healthy pregnant women	Galactooligosaccharides (GOS) and fructooligosaccharides (FOS)	Influence on alpha diversity, influence on F/B ratio No significant change in *Lactobacillus* abundance, increase in *Bifidobacterium* abundance, decrease in Negativicutes abundance, significant decrease in Verrucomicrobiota and Akkermansia Increase in SCFA concentration

**TABLE 2:** Animal studies of maternal prebiotic/probiotic supplementation and its primary maternal outcomes.

References	Animal model	Intervention	Clusters	Primary maternal outcome
Miao et al. (2022)	C57BL/6 mice	Inulin-based fructans (ITF)	Control group (HFD) vs. intervention group (HFD + ITF)	Lower body weight during pregnancy Significant improvement in metabolic parameters, with significant improvement in OGTT AUC, FBG and FSI Increased abundance of Verrucomicrobia, *Bifidobacterium*, and Akkermansia and decreased abundance of Dubosiella Increased levels of acetic acid and butyric acid
Zhang Q. et al. (2019)	C57BL/6 mice	Inulin	Control diet (CD) group vs. HD group vs. intervention (HD + inulin) group	Reduce GWG and FBG
Castro-Rodríguez et al. (2020)	Wistar rat	*Leuconostoc* SD23	Control Diet vs. High Energy Diet vs. Control Probiotics vs. High Energy Probiotics	Improved FBG and HOMA-IR and reduced adiposity in obese rats No significant effect on body weight
Guo et al. (2018)	C57BL/6 mice	*B. breve* DM8310, *L. acidophilus* DM8302, *L. casei* DM8121 and *S. thermophilus* DM8309	Regular diet group vs. high fat diet group vs. intervention group (HFD + probiotics)	Decrease GWG, insulin and leptin levels, no significant changes in FBG Bacteroidetes S24-7, Allobaculum and Sutterella increased in abundance and *Lachnospiraceae*, *Bacteroides*, *Prevotella*, *Mucispirillum*, *Helicobacter*, *Rikenellaceae*, and *Ruminococcaceae* decreased in abundance
Paul et al. (2016)	Sprague-Dawley rat	Oligofructose	Weight-matched group (restricted high-fat diet) vs. high-fat diet group vs. intervention group (high-fat diet + oligofructose)	Reduce GWG No significant differences in blood glucose and insulin levels Higher relative abundance of *Bifidobacteria* and *Bacteroides/Prevotella* spp. Increased levels of SCFA
Brosseau et al. (2021)	BALB/cJRj mice	Galacto-oligosaccharides and inulin	Control group (placebo) vs. intervention group (galacto-oligosaccharide + inulin)	No effect on maternal body weight Significant differences in gut microbiota β-diversity, with increased relative abundance of Muribaculaceae, decreased relative abundance of Desulfobactria and Firmicutes, and remodeling of *Lachnospiraceae* Increased concentration of SCFA
Gao T. et al. (2024)	Sow (long white pig)	*Lactobacillus rhamnosus* GG (LGG)	Control group (regular diet) vs. intervention group (LGG supplementation)	Increased insulin sensitivity during late pregnancy and lactation Significant increase in α-diversity of the gut microbiota, with increased abundance of *Lactobacillus*, *Bacteroides*, and *Methanobrevibacter*, and decreased *Firmicutes/Bacteroidota*
Zhang Z. et al. (2019)	C57BL/6 mice	Lactose (LAC)	Regular diet group vs. 5%LAC group vs. 10%LAC group vs. 15%LAC group	No significant change in GWG Reduce FBG Increase the abundance of Bacteroides and bifidobacteria Increase fecal SCFA abundance

Interestingly, in several clinical studies conducted among pregnant women with high risk of GDM (such as overweight and obesity), probiotic supplementation has consistently demonstrated no significant reduction in GDM incidence (Asgharian et al., 2020; Davidson et al., 2021; Halkjær et al., 2020; Masulli et al., 2020; Pellonperä et al., 2019; Shahriari et al., 2021). This contradiction shows that probiotic intervention alone may not be enough to reverse the pathophysiological process of GDM, so it can only serve as an adjunctive approach for modulating metabolic dysregulation rather than a preventive drug against high-risk state. In addition, a meta-analysis revealed that it may increase the potential risk of preeclampsia (Davidson et al., 2021).

### 3.2 Maternal prebiotic and probiotic intervention regulate gestational body weight gain

Excessive gestational weight gain represents a significant modifiable risk factor for GDM, especially for obese pregnant women. The efficacy of probiotic and prebiotic supplementation for regulating maternal gestational weight is generally limited. In clinical studies, only two reported the possible positive regulatory effect (Dolatkhah et al., 2015; Halkjær et al., 2020), and the rest have no significant effect (Asgharian et al., 2020; Callaway et al., 2019; Jafarnejad et al., 2016; Kijmanawat et al., 2019; Lindsay et al., 2015; Mu et al., 2023; Table 1). However, In studies employing animal models, the use of prebiotic or probiotic could reduce the weight gain during pregnancy in obese individuals (Guo et al., 2018; Miao et al., 2022; Paul et al., 2016; Zhang Q. et al., 2019) or decrease their body fat content (Castro-Rodríguez et al., 2020; Table 2).

### 3.3 Maternal prebiotic and probiotic interventions regulate the gut microbiota

In terms of gut microbiota, probiotic and prebiotic supplementation can substantially modify the composition and function of maternal gut microbiota, manifested by increased α-diversity indices (Halkjær et al., 2020), reduced *Firmicutes/Bacteroidetes* ratio (Jones et al., 2024), elevated abundance of beneficial bacteria [such as *Bifidobacterium* (Halkjær et al., 2020; Jinno et al., 2017; Jones et al., 2024; Paul et al., 2016; Shadid et al., 2007; Zhang Z. et al., 2019) and *Akkermansia* (Miao et al., 2022)], and abundance of potential pathogenic bacteria such as *Dubosiella* (Brosseau et al., 2021; Chen et al., 2019; Guo et al., 2018; Jones et al., 2024; Miao et al., 2022; Table 1). Animal model studies further confirmed this finding (Gao T. et al., 2024). This optimized microbial architecture establishes an ecological foundation for its metabolic benefits.

### 3.4 Effects of prebiotic and probiotic interventions on glucose metabolism in healthy subjects during pregnancy

There are few studies on probiotic or prebiotic supplementation of healthy people, and their results are inconsistent. Partial studies have shown that probiotic supplementation can improve FSI and HOMA-IR (Jamilian et al., 2016; Zheng et al., 2018). In contrast, another study using GOSs did not observe significant effects (Wan et al., 2023). However, in animal models, probiotic or prebiotic supplementation can improve the glucose metabolism of healthy pregnant rodents, reduce blood glucose levels (Zhang Z. et al., 2019) and improve insulin sensitivity (Gao T. et al., 2024). To further determine the glycemic effects of probiotic and prebiotic supplementation in healthy gravidas, it is necessary to conduct a larger sample size study and unify the types, doses and intervention times of probiotics or prebiotics to reduce heterogeneity.

## 4 Effects of maternal probiotic and prebiotic supplementation on offspring

### 4.1 Prebiotic and probiotic intervention influences birth weight and early growth of offspring

Clinical studies generally indicate that probiotic and prebiotic supplementation has no effect on the anthropometry of infants at birth, whether in healthy individuals (Palmer et al., 2025; Wan et al., 2023) or overweight or GDM population (Asgharian et al., 2020; Kijmanawat et al., 2019; Sahhaf Ebrahimi et al., 2019; Shahriari et al., 2021), and has no significant effect on the hypoglycemia rate of newborns (Kijmanawat et al., 2019). Interestingly, healthy women who were supplemented with GOS and FOS during pregnancy had offspring with higher body weights at 6 months (Jones et al., 2024), suggesting that prebiotics may improve the growth performance of offspring (Table 3).

**TABLE 3:** Clinical studies of maternal prebiotic/probiotic supplementation and its effects on offspring.

References	Study design	Subject population	Intervention	Primary offspring results
Wu et al. (2024)	Systematic review and meta-analysis	GDM women	Probiotics	Significant reduction in birth weight
Kijmanawat et al. (2019)	RCT	GDM women	*Bifidobacterium* and *Lactobacillus*	No significant difference in birth weight and neonatal hypoglycemia rate
Sahhaf Ebrahimi et al. (2019)	RCT	GDM women	L. acidophilus and B. lactis	Significant reduction in birth weight
Asgharian et al. (2020)	RCT	Overweight and obese pregnant women	*Lactobacillus acidophilus* LA5 and *Bifidobacterium lactis* Bb12	No significant difference in birth weight
Shahriari et al. (2021)	RCT	Pregnant women in GDM high risk	*Lactobacillus acidophilus* LA1, *Bifidobacterium longum* sp54 cs, and *Bifidobacterium* sp9 cs	No significant difference in birth weight
Palmer et al. (2025)	RCT	Healthy pregnant women	Galactooligosaccharides and fructooligosaccharides	No significant difference in birth weight Decrease in Negativicutes abundance
Wan et al. (2023)	RCT	Healthy pregnant women	Galactooligosaccharides	No significant difference in birth weight
Jones et al. (2024)	RCT	Healthy pregnant women	Galactooligosaccharides and fructooligosaccharides	Higher weight at 6 months Increase α-diversity of gut microbiota and abundance of Bifidobacterium Increase SCFA concentration
Dawson et al. (2024)	RCT	Healthy pregnant women	Probiotics/fermented foods, prebiotics	Reduce α-diversity of gut microbiota

### 4.2 Programing effects of prebiotic or probiotic supplementation on metabolic health of offspring

At present, there is relatively little research on the health of offspring in humans, but in animal experiments, the metabolic benefits of probiotic and prebiotic interventions on offspring are clearer, providing a mechanistic perspective for understanding their intergenerational effects (Table 4).

**TABLE 4:** Animal studies of maternal prebiotic/probiotic supplementation and its effects on offspring.

References	Animal model	Interventions	Clusters	Primary offspring results
Sáez-Fuertes et al. (2024)	Lewis rat	*Bifidobacterium breve* M-16V and short-chain galacto-oligosaccharide (scGOS) and long-chain fructo-oligosaccharide (lcFOS)	Control group (placebo) vs. intervention group (synbiotic supplement)	No significant difference in birth weight and adipose tissue content The α-diversity of cecal microbiota increased significantly, and the abundance of *Bifidobacterium*, *faecalibaculum*, prevotellaceae_ucg_001 increased Increased SCFA content
Luo et al. (2023)	BALB/c mice	*Bifidobacterium infantis* 79 (B79), 2’-fucosyllactose (2’-FL)	Control group (regular diet) vs. probiotic group (B79) vs. prebiotic group (2 ‘ - FL) vs. symbiotic group (B79 + 2 ’ - FL)	No significant difference in weight between 21 and 56 days old The α-diversity of gut microbiota increased significantly, and the abundance of Bifidobacteria increased
Le Bourgot et al. (2024)	Large White × Landrace sow	Short chain fructooligosaccharides (scFOS)	Control group (regular diet) vs. intervention group (scFOS)	No significant difference in birth weight Prevotella abundance increases, Bacteroides abundance decreases SCFA concentration increases
Law et al. (2024)	Mixed hybrid sow	*Saccharomyces cerevisiae* Sc47	Control group (regular diet) vs. 0.1% yeast group vs. 0.5% yeast group	No significant difference in birth weight, but the high yeast diet group shows a decrease in infant weight during infancy Prevotella abundance increases, Catenisphaera and Bacteroides abundance decreases
He et al. (2024)	White land hybrid sow	Pectin (PEC)	Control group (regular diet) vs. intervention group (pectin supplementation)	No significant difference in birth weight The diversity of gut microbiota increases, the relative abundance of Proteobacteria decreases, and the relative abundance of Firmicutes increases
Gao T. et al. (2024)	White land hybrid sow	*Konjac glucomannan* (KGM)	Control group (regular diet) vs. intervention group (KGM supplementation)	No significant difference in birth weight SCFA concentration increases
Djekkoun et al. (2023)	Wistar rat	Inulin	Standard diet group vs. inulin group vs. HFD group vs. HFD + inulin group vs. HFD + CPF group vs. HFD + CPF + inulin group	No significant difference in birth weight Blood sugar level decreases Abundance of Lactobacilli increases
Mohammed et al. (2022)	Golden Syrian hamster	Fructooligosaccharide (FOS)	Control diet (CD) group vs. HFD group vs. HFD + FOS group	No significant difference in weight, Body fat content and body fat accumulation decrease FBG level decreases
Miao et al. (2022)	C57BL/6 mice	Inulin type fructooligosaccharides (ITF)	Control group (HFD) vs. intervention group (HFD + ITF)	Birth weight decreases
Zhang Q. et al. (2019)	C57BL/6 mice	Inulin	Control group (CD) vs. HD group vs. intervention group (HD + inulin)	Birth weight decreases FBG, FSI, and HOMA-IR decrease The abundance of bifidobacteria increases, the abundance of Proteobacteria decreases, and the abundance of butyrate producing bacteria increases
Castro-Rodríguez et al. (2020)	Wistar rat	*Leuconostoc* SD23	Control diet group vs. high-energy diet group vs. control probiotic group vs. high-energy probiotic group	No significant difference in birth weight
Gao T. et al. (2024)	Sow (Landrace Pig)	*Lactobacillus rhamnosus* GG (LGG)	Control diet group vs. intervention group (LGG supplementation)	Relative abundance of beneficial bacteria NK4A214_ group increases and relative abundance of harmful bacteria such as Streptococcus and Klebsiella decreases
Guo et al. (2018)	C57BL/6 mice	*B. breve* DM8310, *L. acidophilus* DM8302, *L. casei* DM8121 and *S. thermophilus* DM8309	Regular diet group vs. high fat diet group vs. intervention group (HFD + probiotics)	Birth weight decreases No significant differences in plasma levels of fasting glucose, insulin, and leptin at birth, decreased leptin levels in adulthood, and decreased glucose and insulin levels in female adult pups Firmicutes abundance decreases, Prevotella abundance decreases, and percentage of gut microbiota reverses
Paul et al. (2016)	Sprague-Dawley rat	*Oligofructose*	Weight-matched group (restricted high-fat diet) vs. high-fat diet group vs. intervention group (high-fat diet + oligofructose)	Body weight, fat mass and percent body fat decrease at 14 days postpartum FPG decreases, no significant difference in insulin levels Abundance of *Bifidobacteria*, *C. coccoides* and *Enterobacteriaceae* increases, abundance of *C. leptum* decreases
Maragkoudaki et al. (2020)	Mice	*Polydextrose* (PDX)	Control diet group vs. obesity diet group vs. obesity diet + PDX group	Weight loss at 6 months with less white adipose tissue and more brown adipose tissue Glucose tolerance improves
Ma et al. (2022)	Bama Mini Pig	*Lactobacillus plantarum* B90 and *Saccharomyces cerevisiae* P11	Control diet group vs. probiotic group	α-diversity of gut microbiota decreases, relative abundance of Deferribacteres and Fusobacteria increases, and relative abundance of Actinobacteria, anaerobes, and rc4-4 decreases
Correia Gomes et al. (2024)	Wistar rat	*L. rhamnosus* GG	Control diet group vs. high calorie diet group vs. control + probiotic group vs. high calorie + probiotic group	No significant difference in birth weight, weight of visceral adipose tissue decreases Blood glucose levels decrease and glucose tolerance increases Abundance of Lactobacillus and Alloprevotella increases, Prevotella abundance decreases

#### 4.2.1 Maternal prebiotic and probiotic interventions alleviate long-term obesity

In animal models fed a normal diet, probiotic or prebiotic supplementation had no effect on offspring body weight (He et al., 2024; Law et al., 2024; Le Bourgot et al., 2024; Luo et al., 2023; Sáez-Fuertes et al., 2024). In contrast, in animal models fed a high-fat/high-sucrose diet, the intervention can reduce progeny body weight later after birth (Guo et al., 2018; Miao et al., 2022; Zhang Q. et al., 2019) and body fat content (Correia Gomes et al., 2024; Maragkoudaki et al., 2020; Mohammed et al., 2022; Paul et al., 2016), and increase the generation of brown adipose tissue that promotes thermogenesis (Maragkoudaki et al., 2020). In conclusion, probiotic or prebiotic supplementation may alleviate obesity and improve the metabolic health of offspring.

#### 4.2.2 Maternal prebiotic and probiotic interventions improve glucose metabolic homeostasis in offspring

**FIGURE 1:**
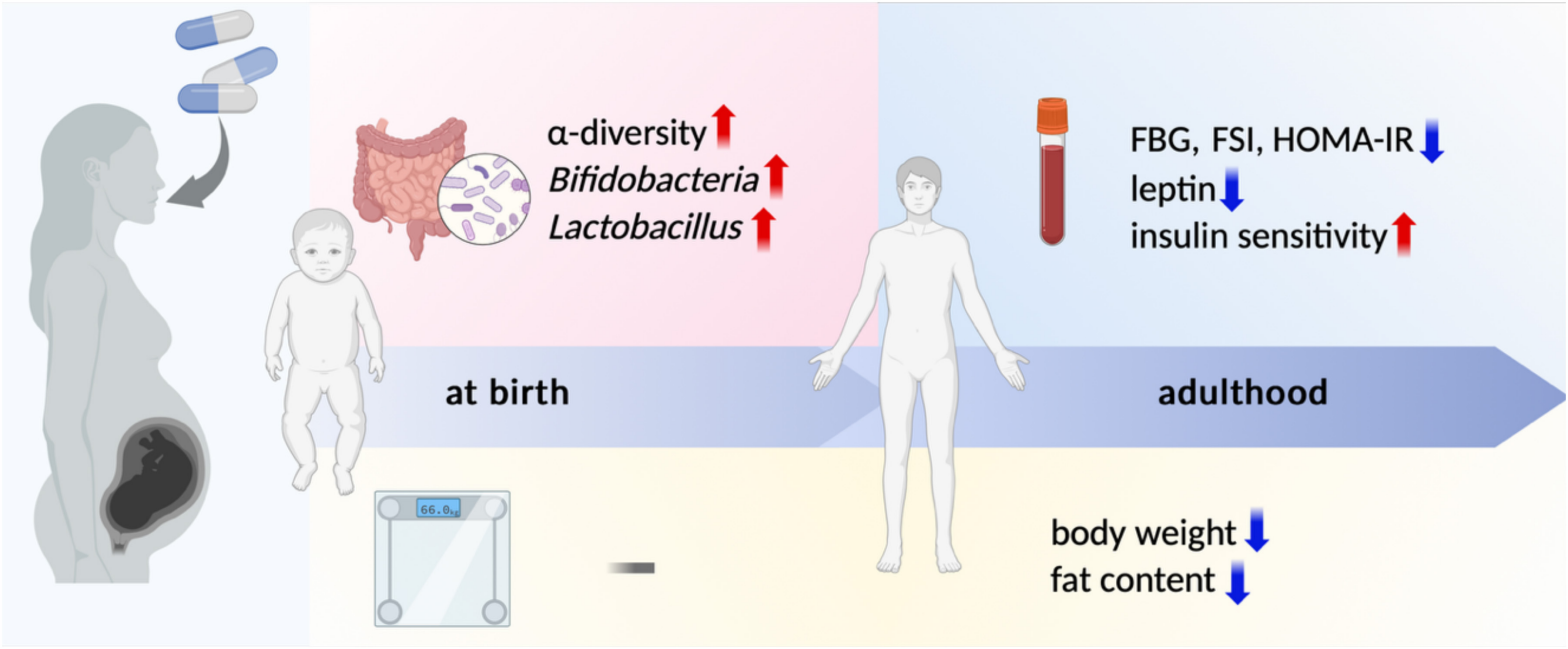
Main effects of maternal probiotic/prebiotic supplementation on offspring. Maternal probiotic or prebiotic supplementation during pregnancy exerts lasting effects on offspring into adulthood. At birth, the primary impacts are observed in alterations of the gut microbiota, such as increased α-diversity and elevated abundances of *Bifidobacteria* and *Lactobacillus*, with no significant effect on birth weight. In adulthood, offspring exhibit improved serum metabolic parameters, including reductions in FBG, FSI, HOMA-IR, and leptin levels, alongside enhanced insulin sensitivity. Furthermore, probiotic or prebiotic supplementation reduces body weight and fat content in adult offspring. FBG, fasting blood glucose; FSI, fasting serum insulin; HOMA-IR, homeostatic model assessment of insulin resistance. Created with BioRender.com.

The results of several studies have shown that probiotic or prebiotic supplementation improves metabolic parameters in the offspring of animals on a high-fat diet, including lowering FBG, FSI, and HOMA-IR and moderating glucose intolerance and insulin resistance (Correia Gomes et al., 2024; Djekkoun et al., 2023; Maragkoudaki et al., 2020; Mohammed et al., 2022; Paul et al., 2016; Zhang Q. et al., 2019). In addition, studies suggest that the metabolic improvement effect can persist into adulthood and is more significant in female offspring (Guo et al., 2018; Figure 1).

#### 4.2.3 Maternal prebiotic and probiotic interventions shape the offspring’s gut microbiota

Probiotic or prebiotic supplementation induces persistent modifications in offspring gut microbial ecology, manifested by an increased α-diversity indices (He et al., 2024; Luo et al., 2023), increased colonization of commensal genera (*Bifidobacteria* and *Lactobacillus* (Correia Gomes et al., 2024; Djekkoun et al., 2023; Luo et al., 2023; Paul et al., 2016; Sáez-Fuertes et al., 2024)), and suppression of potentially pathogenic taxa (*Proteobacteria*, anaerobic bacteria, *Streptococcus*, and *Klebsiella* (Gao T. et al., 2024; He et al., 2024; Ma et al., 2022; Zhang Q. et al., 2019)). Interestingly, in animal models fed a regular diet, probiotic or prebiotic supplementation led to consistent microbial shifts in offspring, marked by elevated *Prevotella* colonization concomitant with reduced *Bacteroides* abundance (Law et al., 2024; Le Bourgot et al., 2024; Sáez-Fuertes et al., 2024), whereas this result was reversed in animal models fed a high-fat diet (Guo et al., 2018). Furthermore, the changes in *Firmicutes* abundance were also the opposite: increasing in the offspring of normally fed animals (He et al., 2024) and decreasing with high-fat diet feeding (Correia Gomes et al., 2024; Guo et al., 2018; Zhang Q. et al., 2019). This may be because obesity itself leads to disruption of the gut microbiota, and probiotic or prebiotic supplementation may reverse this imbalance and promote the construction of a balanced gut microbiota in offspring. In addition, a study partly suggests that the metabolic benefits of maternal probiotic supplementation on offspring can be sustained into adulthood, but is only significant in female offspring (Guo et al., 2018). The reasons for the sex differences remain to be further investigated.

Moreover, probiotic or prebiotic supplementation leads to an increase in SCFA levels in offspring (Gao T. et al., 2024; Le Bourgot et al., 2024; Sáez-Fuertes et al., 2024). This microbial metabolite-mediated mechanism potentially explains the observed metabolic improvements.

## 5 Possible mechanisms of intergenerational effects of maternal prebiotic and probiotic interventions on offspring glucose metabolism

The protective effects of maternal probiotic and prebiotic supplementation on the glucose metabolism of offspring do not stem from the linear regulation of a single pathway; rather, they are achieved through a multi-tiered, spatiotemporal network regulation system encompassing “gut microecology-metabolites-immune inflammation-epigenetics.” This system initiates with the maternal intestinal microbiota as the intervention starting point, proceeds via signal transduction mediated by metabolites (e.g., SCFAs, secondary BAs) and systemic alleviation of inflammatory states, and subsequently “inscribes” metabolic information into the offspring’s genome through epigenetic mechanisms such as DNA methylation and non-coding RNAs, ultimately reshaping the glucose metabolism homeostasis of the offspring. This mechanistic framework not only elucidates the intergenerational transmission pathway of maternal microecological interventions but also provides actionable molecular targets for understanding the DOHaD hypothesis.

### 5.1 Mother-offspring vertical transmission of the gut microbiota composition

According to the studies summarized in the previous section, probiotic or prebiotic supplementation of the mother can induce modifications in the gut microbiota of offspring by exerting metabolic benefits. Since the main effect of probiotic or prebiotic supplementation is observed in the metabolic improvement of offspring in animal models fed a high-fat diet, the following text will emphasize the effects of probiotic or prebiotic supplementation of obese mothers on the gut microbiota composition of offspring.

As mentioned previously, gut microbial dysbiosis is intrinsically linked to metabolic disease pathogenesis in obesity and diabetes. Moreover, maternal gut microbial perturbations can be vertically transmitted to offspring (Collado et al., 2010; Galley et al., 2014); therefore, maternal microbial dysbiosis compromises the establishment of offspring gut microbiota, subsequently inducing detrimental effects on glucose homeostasis.

Probiotic and prebiotic supplementation can regulate the imbalance of the gut microbiota and normalize the elevated F/B ratio, thereby improving glucose metabolism. These microbial-targeted nutritional interventions also consistently promote the colonization of commensal microorganisms (e.g., *Bifidobacterium*, *Lactobacillus*, *Akkermansia*, etc.) while simultaneously suppressing enteric pathobionts (particularly *Enterobacteriaceae*). *Bifidobacteria* and *Lactobacillus* have been recognized for their antiobesity and metabolic-improving effects and are widely used because of their safety and antibiotic resistance, leading to variable reductions in body weight and fat accumulation, enhancement of glycemic control and improvement of insulin sensitivity (Li Y. et al., 2024; Lyu et al., 2024). There is evidence that *Bifidobacteria* and *Lactobacillus* are negatively correlated with insulin and HOMA-IR (Tabasi et al., 2021; Teixeira et al., 2013; Wang Y. et al., 2023; Zhang et al., 2024) and are important targets for modulating the gut microbiota to improve glucose metabolism, which can improve glucose homeostasis by increasing GLP-1 expression levels and decreasing intestinal permeability (Rodes et al., 2013; Song H. et al., 2023). *Lactobacillus* can also increase antioxidant enzyme activity to increase oxidative stress capacity (Amaretti et al., 2013; Song H. et al., 2023), thereby reducing body fat content and improving glucolipid metabolism. *Akkermansia* is also recognized as a key bacterium in glucose metabolism, with a positive correlation between its abundance and the metabolic health of the host (Shin et al., 2014). *Akkermansia* improves intestinal mucosal barrier function, reduces insulin resistance and intestinal inflammation (Plovier et al., 2017), and secretes GLP-1-inducible proteins (Yoon et al., 2021) to promote glucose homeostasis.

In conclusion, probiotic and prebiotic supplementation improves offspring glucose metabolism by modulating intestinal microbial ecosystems which influences both maternal and fetal microbiome development.

### 5.2 Metabolic delivery of metabolites from the intestinal flora

#### 5.2.1 Short chain fatty acids

Accumulating evidence indicates that probiotic and prebiotic supplementation can enhance populations of butyrate-producing bacteria, consequently increasing the concentration of gut metabolites such as SCFAs to improve glucose metabolism. There is evidence that probiotic or prebiotic supplementation leads to an increase in the concentration of SCFAs in offspring, suggesting that SCFAs may play a key role in improving glucose metabolism in offspring by altering the gut microbiota. First, SCFAs function as potent microbial-derived signaling molecules that activate G protein-coupled receptors (GPR41/GPR43) to stimulate GLP-1 and PYY secretion (Psichas et al., 2015; Tolhurst et al., 2012), which enhances glucose uptake in muscle and adipose tissues, increases satiety, reduces food intake, and stimulates leptin secretion in adipocytes, thereby reducing appetite (Chambers et al., 2015). It induces sympathetic activation via GPR41 to control body energy expenditure to maintain metabolic homeostasis (Kimura et al., 2011). Second, SCFAs upregulate GLUT2 mRNA expression, thereby maintaining glucose homeostasis (Mangian and Tappenden, 2009). Third, SCFAs serve as the primary energetic substrate for colonic enterocytes and maintain cholesterol-rich microstructural domains in the colonic epithelial plasma membrane to reduce intestinal permeability and enhance intestinal barrier function (Parada Venegas et al., 2019). In addition, SCFAs may also improve glucose metabolism by modulating multiple inflammatory mediators and reducing proinflammatory responses (Yu et al., 2025).

#### 5.2.2 Secondary bile acids

BAs represent crucial microbial metabolites whose biosynthesis involves a complex hepatointestinal–microbial axis. Specifically, the gut microbiota mediates BA transformation through four principal enzymatic modifications: amino acid deconjugation (glycine/taurine cleavage), steroid nucleus 7α-dehydroxylation, dehydrogenation of hydroxyl groups, and stereospecific epimerization (Guzior and Quinn, 2021). BAs exert pleiotropic metabolic effects through the activation of both nuclear and membrane-bound receptors, with the key receptors involved in glucose metabolism being the nuclear receptor FXR and the membrane receptor TGR5 (Gao et al., 2022). On the one hand, BAs improve glucose sensitivity and reduce insulin resistance by activating FXR via the SHP-dependent and FGF15/19-mediated pathways (Kong et al., 2012; Sun et al., 2018; Wang Y. et al., 2023). On the other hand, secondary BAs demonstrate 3—5-fold greater TGR5 binding affinity than primary BAs do. BAs can induce GLP-1 release through activation of the TGR5 receptor (Wang Q. et al., 2023; Zheng et al., 2021). Furthermore, TGR5 induces the upregulation of CKMT2 and UCP1 in mouse inguinal brown adipose tissue via the cAMP—PKA pathway, which increases oxidative phosphorylation and energy expenditure and controls thermogenesis (Chen et al., 2023; Wu et al., 2021).

### 5.3 Mechanisms by which maternal prebiotic and probiotic intervention affects offspring glucose metabolism through inflammation

An abnormal inflammatory state in the body promotes the proliferation of proinflammatory cytokines (TNF-α and IL-6), which activate signaling pathways such as PKC and mTOR/S6K leading to systemic insulin resistance (Saad et al., 2016). There is evidence that maternal probiotic supplementation decreases plasma IL-1β, IL-2, IL-6, and IFN-α levels (Hajifaraji et al., 2018; Ma et al., 2022); similarly, prebiotic supplementation suppresses the expression of the genes MCP-1, IL-6, and IL-1β (Paul et al., 2019). These results suggest that probiotic and prebiotic supplementation can attenuate offspring inflammatory responses through the downregulation of proinflammatory cytokine expression and that the observed decrease in inflammatory mediator levels is correlated with improved insulin sensitivity. In addition, a study by Mohammed et al. revealed that maternal FOS supplementation reduced neutrophil infiltration of the placenta, decreased high-fat diet-induced placental and intrauterine inflammation and ameliorated the adverse intrauterine environment (Mohammed et al., 2022). Prebiotic supplementation also reduced leptin mRNA levels in offspring (Paul et al., 2019) and had a protective effect on glucose metabolism.

### 5.4 Mechanisms by which maternal prebiotic and probiotic interventions affect offspring glucose metabolism through epigenetics

Epigenetics refers to the induction of shifts in gene expression patterns without altering the structure of the DNA sequence and its mechanically mediated through covalent DNA methylation, posttranslational histone modification and non-coding RNA-mediated silencing complexes (Ling and Rönn, 2019). On the basis of the available evidence, epigenetic inheritance is a possible mechanism by which the gut microbiota regulates glucose metabolism in offspring. There are few studies on the epigenetic mechanisms of probiotic and prebiotic supplementation, including the following possible modes of regulation.

#### 5.4.1 DNA methylation

DNA methylation is a covalent biochemical modification involving the enzymatic transfer of a methyl group (-CH3) to the fifth carbon position of cytosine residues (Elhamamsy, 2016). Typically, methylation of the promoter region of a gene inhibits gene transcription, thereby decreasing gene expression (Yin et al., 2017). Zhang et al. (2020) reported that maternal prebiotic inulin supplementation of high-fat diet-fed mice increased the methylation of the Wnt5a gene in the livers of offspring, which in turn decreased the expression of the Wnt5a gene. The Wnt5a gene is closely related to metabolic inflammation in humans (Relling et al., 2018) and can activate c-Jun N-terminal kinase 1 (JNK1) in adipocytes through the β-catenin-independent signaling pathway, thereby blocking insulin receptor substrate-1 (IRS-1) activity, leading to impaired insulin receptor signaling cascade efficiency and promoting the development of insulin resistance (Farb et al., 2016; Koutaki et al., 2021). Inulin supplementation also inhibits PI3K methylation, activating its expression. The PI3K—AKT signaling pathway is a major effector molecule of insulin action (Schultze et al., 2012), and activation of this pathway improves insulin sensitivity and thus glucose metabolism. A randomized controlled study (Vähämiko et al., 2019) conducted in the Netherlands revealed that *Lactobacillus ramosus GG* and *Bifidobacterium lactis Bb12 (C.)* supplementation significantly reduced the level of promoter DNA methylation of genes related to glucose metabolism and obesity, including insulin-like growth factor-binding protein 1 (IGFBP1) and methionine sulfoxide reductase A (MSRA) genes, in offspring. IGFBP1 can bind to IGF-1 and competitively inhibit its interaction with cell surface receptors (Jones and Clemmons, 1995), thereby affecting signaling in the insulin signaling pathway and regulating insulin concentrations. MSRA has been shown to reduce oxidized methionine residues, thereby participating in protein repair and protection from oxidation. In conclusion, maternal probiotic or prebiotic supplementation may regulate glucose metabolism by altering the DNA methylation status of offspring.

#### 5.4.2 lncRNAs

LncRNAs are non-coding RNAs of approximately 200 amino acids in length that regulate transcription, epigenetic modifications, translation and posttranslational modifications and play important roles in many cellular processes (Bridges et al., 2021). Currently, increasing evidence indicates that lncRNAs have key roles in metabolic diseases such as diabetes. Zhang et al. (2021) supplemented high-fat diet-fed C57BL6/J mice with inulin throughout gestation and lactation and analyzed the differential expression of lncRNAs and the pathways involved in their male offspring. The results revealed that maternal supplementation with probiotic inulin resulted in the differential expression of 99 lncRNAs and 529 mRNAs, and the main pathway affected was the AMPK signaling pathway, with the key gene being Hnf4a. In addition, inulin inhibited the function of the hepatic Serpina4-ps1/let-7b-5p/Ppargc1a axis, which can activate the PGC-1α protein, a master transcriptional coactivator that coordinately regulates mitochondrial biogenesis, fatty acid oxidation, peroxisome biogenesis and carbohydrate metabolism (Aisyah et al., 2022; Liu and Lin, 2011; Prior et al., 2012). In conclusion, maternal inulin supplementation may have a protective effect on insulin sensitivity and glucose metabolism in offspring through changes in lncRNA expression.

#### 5.4.3 miRNAs

MiRNAs are short RNAs of approximately 22 amino acids in length that function to posttranscriptionally regulate gene expression through complementary base—pairing with target mRNAs. Human breast milk contains substantial quantities of miRNAs, which are mostly encapsulated within a lipid bilayer and transported by exosomal vesicles, conferring protection against enzymatic degradation. Therefore, breast milk-derived miRNAs are biologically active in offspring and are capable of entering the progeny cycle and altering gene expression through epigenetic mechanisms. In other words, miRNAs could constitute another possible epigenetic mechanism for regulating glucose metabolism in offspring. Lowry et al. (2022) provided prebiotic oligofructose (OFS) to SD rats fed a high-fat diet and measured miRNA levels in their milk. The results showed that OFS supplementation decreased the miR-222 and miR-200a levels in the rats’ milk. MiR-222 levels are elevated in obese and overweight individuals. In addition, increased miR-222 levels were detected in T2DM patients. Therefore, miR-222 is a potential biomarker for the diagnosis of T2DM (Sadeghzadeh et al., 2020). There is evidence that miR-222 leads to direct inhibition of the insulin signaling pathway, affects adipocyte differentiation (Bibiloni et al., 2023) and promotes triglyceride accumulation (Wang et al., 2019). MiR-200a has been mechanistically linked to non-alcoholic fatty liver disease (NAFLD), and its expression is increased under inflammatory conditions. Thus, prebiotic OFS supplementation may alleviate insulin resistance and attenuate inflammatory responses by reducing the level of miR-222 and miR-200a, and this effect can be transmitted to offspring through milk. In addition, OFS supplementation reduced milk miR-27a, miR-103, and miR-26a levels, which were previously correlated with offspring metabolic dysfunction (obesity, metabolic syndrome, and T2DM). This study is the first to suggest an effect of prebiotic supplementation on breast milk composition, providing new avenues for understanding the intergenerational effects of prebiotics on glucose metabolism, but it did not measure the levels of miRNAs in offspring to determine their effect on gene expression. Thus, the evidence is incomplete and needs to be supplemented by more subsequent studies. In conclusion, miRNAs are a potential epigenetic mechanism for the regulation of glucose metabolism in offspring by probiotic and prebiotic supplementation and are transmitted to offspring through milk.

## 6 Discussion: controversies, limitations, and future directions

### 6.1 Heterogeneity in efficacy and the dilemma of preventive intervention

A central controversy is the discordance between therapeutic and preventive trials: probiotics and prebiotics consistently improve glvcemic control in established GDM but fail to reduce GDM incidence in high-risk populations. This suggests a threshold effect—intervention restores disrupted homeostasis but confers marginal benefit when metabolic function remains intact. It also questions whether GDM incidence is an overly stringent endpoint; metabolic trajectory may be a more sensitive alternative.

### 6.2 Methodological limitations: revisiting sources of heterogeneity

Beyond population characteristics and intervention objectives, the heterogeneity of current evidence is deeply rooted in methodological shortcomings inherent to study design First, the intervention plan is highly heterogeneous—the lack of uniform standards in various studies on the combination of strains, prebiotic types, doses, initial gestational weeks, and intervention duration lead to the inability to determine the optimal program. Second, the sample size is generally insufficient and rarely includes effect modification factors such as baseline flora composition and dietary fiber intake for hierarchical analysis. Third, the follow-up window is severely limited, and most studies stop at the end of delivery or intervention. The lack of follow-up data in childhood and even adulthood makes it impossible to answer whether maternal intervention can really change the trajectory of diabetes in children.

### 6.3 Causal gaps in mechanistic research

Although the regulatory network of “micreobiota-metabolite-immunity-epigenetics” has been widely described, most links still remain at the correlation level, and the verification of causal chain is seriously insufficient: whether the change of flora and metabolic improvement are driven or accompanied, there is no direct evidence of fecal flora transplantation experiment in the context of GDM pregnancy. The spatiotemporal distribution and receptor expression dynamics of SCFAs and secondary BAs at the pregnancy fetal interface and during fetal organ development have not been systematically mapped. Epigenetic studies are mostly limited to the expression reports of single gene or non-coding RNA, and lack of multi omics integration and functional verification. Whether methylation/non-coding RNA changes really lead to downstream metabolic pathway shift has not been confirmed by most studies.

### 6.4 Future directions

Based on the above limitations, future research should break through the following dimensions:

First, promote the standardization and dose optimization of intervention programs, and provide evidence-based basis for clinical transformation. Second, carry out individualized intervention of flora typing. Classify intestinal types by pre-enrollment bacterial sequencing and match targeted probiotic strains or prebiotics according to the characteristics of specific probiotic deletion or pathogen enrichment. Third, build a long-term birth queue and nested RCT. The follow-up of offspring was extended to school age and adolescence, including refined phenotypes such as glucose tolerance, body composition and insulin secretion, to discover the persistence of metabolic programing effect. Fourth, strengthen causal inference and mechanism verification. Use tools such as Mendelian randomization and mediation analysis. In the animal model, carry out both-way verification of function acquisition and deletion of candidate pathways through aseptic colonization, metabolite intervention and gene knockout.

## 7 Summary and outlook

**FIGURE 2:**
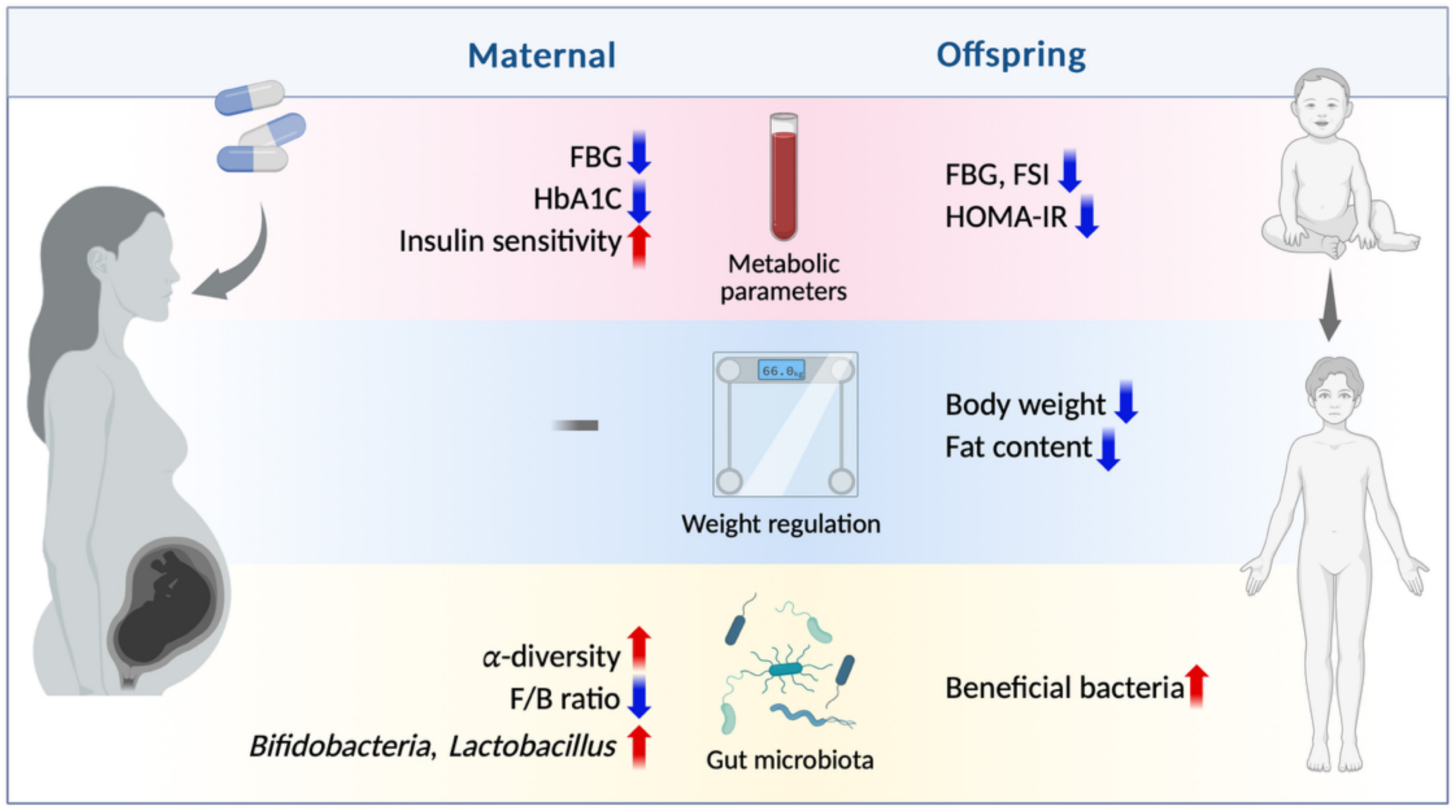
Main effects of maternal prebiotic/probiotic supplementation on pregnant women and offspring. Maternal prebiotic and probiotic supplementation during pregnancy may contribute to glucose metabolism of both mothers and offspring. To mothers, the intervention leads to improvement in metabolic parameters, such as reduced FBG and HbA1c levels, along with enhanced insulin sensitivity. However, there is no significant difference to gestational weight gain. Additionally, it increases gut microbiota α-diversity, restores F/B ratio, and enhances the abundance of *Bifidobacteria* and *Lactobacillus*. To offspring, maternal prebiotic and probiotic supplementation decreases the level of FBG, FSI and HOMA-IR. It also reduces body weight and fat content while promoting the growth of beneficial bacteria in their gut microbiota. FBG, fasting blood glucose; FSI, fasting serum insulin; HOMA-IR, homeostatic model assessment of insulin resistance; F/B ratio, *Firmicutes/Bacteroidetes* ratio. Created with BioRender.com.

**FIGURE 3:**
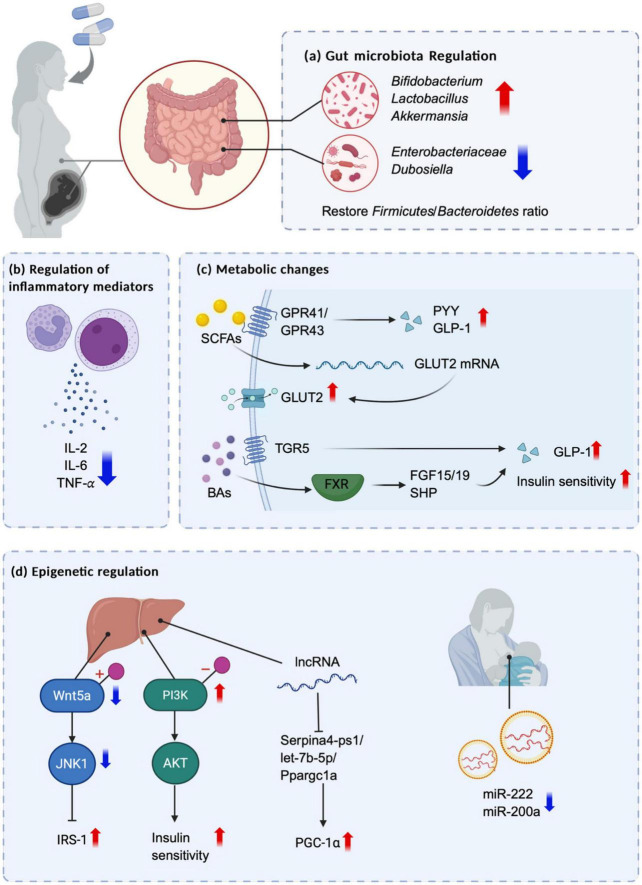
Possible mechanisms of intergenerational effects of maternal prebiotic and probiotic interventions on offspring glucose metabolism. Maternal probiotic or prebiotic supplementation during pregnancy exerts multifaceted effects on offspring through modulation of gut microbiota and its metabolites, regulation of inflammatory mediators, and epigenetic mechanisms. **(a)** It enhances beneficial bacterial populations (e.g., *Bifidobacterium*, *Lactobacillus*, and *Akkermansia*) while reducing potential pathogens (e.g., *Enterobacteriaceae* and *Dubosiella*), accompanied by remodeling of the F/B ratio. **(b)** It ameliorates systemic inflammation by decreasing pro-inflammatory cytokines such as IL-2, IL-6, and TNF-α, thereby contributing to metabolic improvements. **(c)** These metabolic benefits are mediated through microbial metabolites, where SCFAs activate GPR41/43 to stimulate PYY and GLP-1 secretion while upregulating GLUT2 expression, and BAs signal through TGR5 and FXR receptors to enhance GLP-1 release and improve glucose homeostasis via FXR-SHP and FXR-FGF15/19 pathways. **(d)** Epigenetically, maternal intervention modulates DNA methylation patterns, increasing Wnt5a methylation to suppress JNK1-IRS-1 inhibition while decreasing PI3K methylation to activate the PI3K-AKT pathway, collectively improving insulin sensitivity. Additional epigenetic regulation occurs through lncRNA-mediated suppression of the Serpina4-ps1/let-7b-5p/Ppargc1a axis to control PGC-1α expression, and miRNA-dependent mechanisms where reduced breast milk miR-222 and miR-200a levels alleviate offspring insulin resistance. Created with BioRender.com.

Early life is a critical period of human growth and development and is vulnerable to adverse environmental influences. Current research suggests that probiotic and prebiotic supplementation in early life significantly improves glucose metabolism in both mothers and offspring (Figure 2) by mechanisms involving the gut microbiota and its metabolites, inflammation, and epigenetics (Figure 3). Animal experiments provide supportive evidence, but there are inconsistencies in the results of human studies on their metabolic benefits, which may be related to the type of probiotic or prebiotic, the dosage and the timing of the intervention. Additionally, the size of the existing clinical studies is too small to exclude the individual differences in the subject populations; thus, the results still need to be further validated with large-scale and long-term studies. In addition, while the observed parameters of the current clinical studies have focused mainly on the effects on mothers, further research should prioritize longitudinal assessment of perinatal probiotic or prebiotic supplementation on offspring glucose homeostasis to assess its intergenerational effects.

The exploration of the mechanism of probiotic and prebiotic supplementation is still incomplete, highlighting the need for future investigations to elucidate microbiota–methylomic crosstalk and characterize microbial metabolite signaling. Particular emphasis should be placed on mapping tissue-specific metabolic programing pathways and the focal point could be the TLR4/NF-κB and PPARγ transcriptional networks as potential mechanistic hubs. In addition, because of the large individual differences in the gut microbiota of different human bodies, personalized probiotic and prebiotic intervention strategies based on the characteristics of individual gut microbiota could be explored for the implementation of precise and individualized medical treatment.

In summary, on the basis of the known metabolic benefits of probiotics and prebiotics, we recommend that mothers (especially those who are obese or suffer from metabolic diseases) continue to supplement with probiotics and prebiotics throughout pregnancy to improve the metabolic health of both mothers and offspring. Since miRNA alterations resulting from prebiotic supplementation can be transmitted through milk, and since prebiotic supplementation during lactation has been demonstrated to improve offspring microbial colonization in the early postpartum period (Marousez et al., 2023), probiotic and prebiotic supplementation should be sustained throughout lactation. In addition, the safety of probiotic and prebiotic supplementation has been well documented based on available studies, making their use in improving the metabolic health of offspring promising. In the future, probiotic or prebiotic supplementation, along with maternal diet and medication, is expected to become an important method to improve glucose metabolism in offspring for the early prevention and control of metabolic syndromes such as diabetes.
